# Multiplex Immunofluorescence Tyramide Signal Amplification for Immune Cell Profiling of Paraffin-Embedded Tumor Tissues

**DOI:** 10.3389/fmolb.2021.667067

**Published:** 2021-04-29

**Authors:** Sharia Hernandez, Frank Rojas, Caddie Laberiano, Rossana Lazcano, Ignacio Wistuba, Edwin Roger Parra

**Affiliations:** Department of Translational Molecular Pathology, The University of Texas MD Anderson Cancer Center, Houston, TX, United States

**Keywords:** immune microenvironment, multiplex immunofluorescence, immune profiling, cell phenotyping, immunotherapy

## Abstract

Every day, more evidence is revealed regarding the importance of the relationship between the response to cancer immunotherapy and the cancer immune microenvironment. It is well established that a profound characterization of the immune microenvironment is needed to identify prognostic and predictive immune biomarkers. To this end, we find phenotyping cells by multiplex immunofluorescence (mIF) a powerful and useful tool to identify cell types in biopsy specimens. Here, we describe the use of mIF tyramide signal amplification for labeling up to eight markers on a single slide of formalin-fixed, paraffin-embedded tumor tissue to phenotype immune cells in tumor tissues. Different panels show different markers, and the different panels can be used to characterize immune cells and relevant checkpoint proteins. The panel design depends on the research hypothesis, the cell population of interest, or the treatment under investigation. To phenotype the cells, image analysis software is used to identify individual marker expression or specific co-expression markers, which can differentiate already selected phenotypes. The individual-markers approach identifies a broad number of cell phenotypes, including rare cells, which may be helpful in a tumor microenvironment study. To accurately interpret results, it is important to recognize which receptors are expressed on different cell types and their typical location (i.e., nuclear, membrane, and/or cytoplasm). Furthermore, the amplification system of mIF may allow us to see weak marker signals, such as programmed cell death ligand 1, more easily than they are seen with single-marker immunohistochemistry (IHC) labeling. Finally, mIF technologies are promising resources for discovery of novel cancer immunotherapies and related biomarkers. In contrast with conventional IHC, which permits only the labeling of one single marker per tissue sample, mIF can detect multiple markers from a single tissue sample, and at the same time, deliver extensive information about the cell phenotypes composition and their spatial localization. In this matter, the phenotyping process is critical and must be done accurately by a highly trained personal with knowledge of immune cell protein expression and tumor pathology.

## Introduction

Recently, crucial developments in cellular immunology helped facilitate the translation of immunologic concepts into new immunotherapies. In cancer immunotherapies, the immune system is activated to strike tumor cells through natural mechanisms that were lost or evaded during disease progression (Riley et al., 2019). Instead of directly killing cancer cells, these therapies aim to improve antitumor immune responses, with fewer off-target effects than are observed with chemotherapy agents, shifting the cancer treatment paradigm (Mitchison, 1955; Rosenberg, 2014; Parra et al., 2018; Riley et al., 2019).

The tumor microenvironment consists of tumor cells, immune cells, fibroblasts, tumor vasculature, and the extracellular matrix. Their interactions can promote tumor transformation, tumor protection from host immunity, tumor growth, and tumor invasion and can foster therapeutic resistance (Yu and Cui, 2018). To determine the effect of the host immune response to tumor formation and invasion, researchers can analyze immune components and their organization within human tumors. Because immune infiltrates differ between tumor types and even between patients, an analysis of the location, density, and spatial orientation of the different immune cell populations in large annotated collections of human tumors allows for the identification of beneficial immune components, as well as those that might indicate a poor prognosis (Fridman et al., 2012; Pilla and Maccalli, 2018).

An increasing number of studies have characterized immune infiltrates for T-cell subsets, B cells, macrophages, etc., and some studies have also included activation and functional markers (Bethmann et al., 2017). Immune profiling can be achieved through various technologies, such as conventional technologies [e.g., single immunohistochemistry (IHC) and early-generation fluorescence-based flow cytometry] and multiplex technologies (Parra et al., 2016; Taube et al., 2020; Wang et al., 2020). Conventional technologies, such as single IHC, have many limitations, including fewer available analysis parameters, a greater sample quantity requirement, and sometimes overlapping detection signals. The newer and higher-dimensional technologies avoid many of these limitations (Chuah and Chew, 2020).

Over the last years, multiplex techniques are widely defined as technologies used to identify multiple biological markers in different tissue samples (Dixon et al., 2015; Taube et al., 2020). Using these technologies, individual cells can be assessed with extraordinary fidelity, and rare cell populations can be studied, providing unique biological information that, in many cases, cannot be obtained by conventional techniques (Parra et al., 2019a). Multiplex technologies are based on the analysis of the expression of proteins of interest, which correspond to specific cell types and biological processes, providing an insight about cell characteristics and their biological interactions. Additionally, the resulting single-cell data can be analyzed using qualitative and quantitative approaches in the context of the original spatial arrangement of the tissue cells (Rashid et al., 2019). The spatial cell distributions can be analyzed to link their biological interactions with the morphological characteristics of tumoral tissues (Barua et al., 2018). Compared to previous tissue analysis methods, multiplex technologies provide a more comprehensive view of tissue composition and marker distribution (Bodenmiller, 2016).

In this setting, we find multiplex immunofluorescence (mIF) a powerful and useful tool to identify different cell phenotypes in biopsy specimens. In this article, we describe the use of mIF tyramide signal amplification to for immune cell profiling of formalin-fixed, paraffin-embedded tumor tissues.

## Panel Design and Selection

**FIGURE 1:**
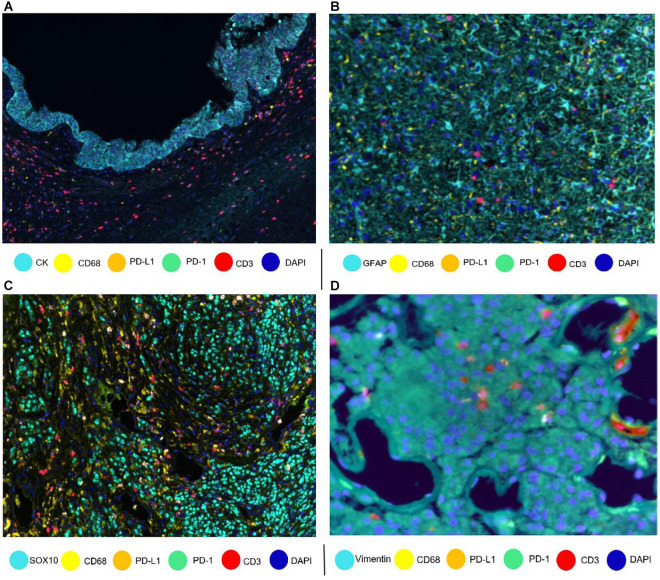
Composites of tumor samples stained with different tumor and epithelium antibodies. **(A)** Intraductal papillary mucinous neoplasm epithelium with cytokeratin (CK). **(B)** Glioblastoma glial cells with glial fibrillar acidic protein (GFAP). **(C)** Melanoma with nuclear SOX10. **(D)** Sarcoma with vimentin.

Designing a mIF panel for a specific project requires selecting and validating appropriate antibodies chosen by a multidisciplinary team of experts in oncology, pathology, and immunology, to ensure that the panel will appropriately address the aims of the project and be able to comprehensively and coherently identify the specific cell phenotypes of interest (Parra et al., 2017, 2020b). Researchers can create panels with groups of markers to study different immune cell populations [using programmed cell death ligand 1 (PD-L1) and programmed cell death 1 (PD-1)], T-cell behavior (using stimulatory and regulatory T-cell markers), and myeloid cell populations (using more targeted panels). Besides, every panel can be customized depending on the type of tumor. For example, cytokeratin antibody can be used as an epithelial tumoral marker (Krishna, 2010), glial fibrillar acidic protein as a glioblastoma marker (Guichet et al., 2016), SOX10/S100 as a melanoma marker (Mohamed et al., 2013), and vimentin as a marker for some sarcomas (Figure 1). We can use different immune markers in the mIF panels to identify more specific phenotypes, such as using TMEM119 to identify microglia in brain tissues (Satoh et al., 2016).

## Tissue Selection

Ideally, formalin-fixed, paraffin-embedded tumor samples should be at least 10 mm × 2 mm, with tumor cells accounting for at least 10% of the biopsy specimen. Furthermore, a threshold of 100 malignant cells identified by markers is considered necessary to minimize the risk of errors in the analysis and interpretation of the samples, as is the case of PD-L1 expression (Tsao et al., 2018). During the analysis, necrotic areas, such as those observed in tumors treated with neoadjuvant therapies, should be excluded, as should material secreted by tumors, such as mucus, that can limit the quality of the analysis, and the results containing these characteristics should be excluded. Thus, a pathology quality assessment is a very important and necessary step for the selection of oncology samples (Parra et al., 2017).

## Tissue and Cell Segmentation for Cell Phenotyping

Overall, the image analysis software, Inform software (Akoya Biosciences), needs to have tools for different purposes, such as tumor compartmentalization. Tumor compartmentalization will depend on the markers included in a panel. For this purpose, and based on the expression or absence of tumor markers (e.g., cytokeratin and SOX10/S100), we can divide the image into tumor cell nests and the stromal compartment (Parra et al., 2020b). The tools need to be flexible enough to identify other compartments, such as vessel areas, necrotic areas, and empty space as glass areas (areas without tissue).

Training a software to individualize the cells is crucial and one of the key steps to obtain accurate data. For this purpose, 4’,6-diamidino-2-phenylindole is useful, and it is used for nuclear quantitation to visualize nuclear DNA in formalin-fixed, paraffin-embedded tissues (Tarnowski et al., 1991). It can be used alone or in combination with membrane markers, such as CD3, or cytoplasmic markers, such as cytokeratin, to better identify and individualize the cells. Modifying parameters, such as nuclear size and nuclear staining thresholds, or using tools that combine such parameters is essential to better identify and individualize cells. Because every tumor and sample are different, adjusting these parameters based on tumor type will probably be necessary (Supplementary Figure 1).

## Importance of Marker Identification

**FIGURE 2:**
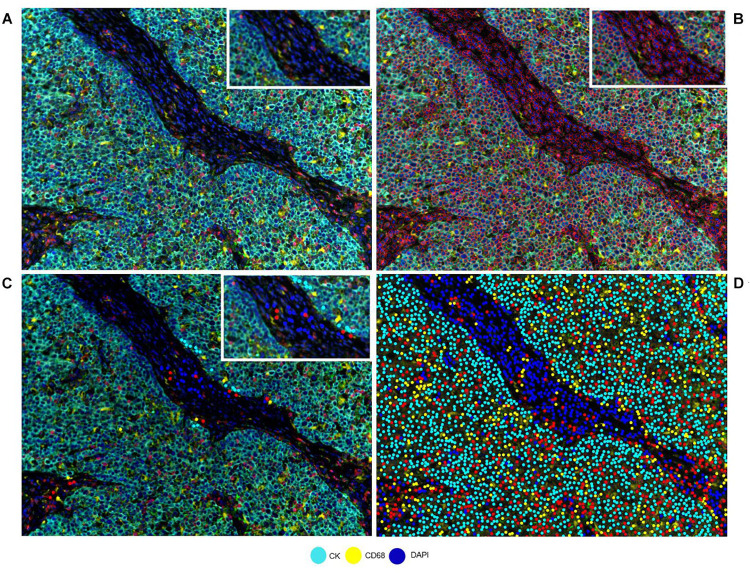
Developing a phenotyping algorithm for an intraductal papillary mucinous neoplasm image. **(A)** Composite image. **(B)** Cell segmentation with red lines surrounding the cells. **(C)** Phenotyping examples. **(D)** Phenotype result of the software after training. White-framed rectangles on the images identify the same area in the four images, and this area has been amplified in the white-framed rectangles on the upper right of each panel.

Correct understanding of the individual markers in a mIF panel is crucial to identify different cell phenotypes in tumor tissues. Identifying individual markers and identification of the combination of different markers at different levels are distinct approaches that have a similar goal: cell phenotyping, or the final identification of the marker’s co-expression by the same cells. Individual markers, such as the ones used in mIF panels, are complex and can be co-expressed in multiple cells. The image analysis tools can facilitate the creation of thresholds for individual markers, based on the pathology visualization and multiple rounds of software training (Figure 2). To create such thresholds, both the morphology of the stained cells and the subcellular compartment that is stained must be considered. As an example, PD-L1 is expressed by the membrane of tumor cells and macrophages, but because lymphocytes are small cells with very scarce cytoplasm that cannot always be distinguished from the cell membrane, we consider strong lymphocyte cytoplasmic and/or membrane expression to be positive expression. As another example, some cells, such as hepatocytes, can constitutionally express arginase-1 (Yan et al., 2010). However, myeloid cells also express arginase-1 (Grzywa et al., 2020), so co-localization of arginase-1 with cytokeratin in hepatocytes or with CD68 in macrophages helps us to identify the cell phenotype of interest. Also, co-localization can help to distinguish between real staining and artifacts or background. If we have doubts in a subset of cells (e.g., some that express CD8, Foxp3, or PD-1, which can all also be expressed by T-cells), we can always visualize the CD3 co-expression to be sure of the marker expression of that specific cell. Nevertheless, negative controls always need to be included to avoid the autofluorescence that certain tissues emit during the preparation of the image and to obtain a clearer signal, taking off any interference of the autofluorescence.

## Marker Consolidation and Assessment of Cell Phenotypes

Because a single cell can express many immune markers, individual marker analysis is usually a very efficient approach and can result in a large variety of cell phenotypes in the consolidation step, which uses consolidation software, such as R-studio (Ye, 2016) and SAS (Dembe et al., 2011). The data need to be placed in a comprehensive table categorizing immune cell phenotype (co-expression of markers) densities or percentages. The data also need to be reviewed and pass a quality control to ensure their accuracy. For example, the total number of cells should be similar to the quantity of cells observed while processing the image samples. We have also found that processing images, while qualitative, allows a pathologist to become familiar with the images and detect “odd” numbers that do not correlate with the nature of cases. When using multiple mIF panels to study samples, it is important to incorporate a common marker as an internal control in each panel. For example, CD3 is usually used in different mIF panels to study lymphocyte subpopulations. Although different levels of the formalin-fixed, paraffin-embedded biopsy specimens are used, we always try to obtain close cut levels during the staining process of the sample to achieve similar cellularity between panels. This goal makes it possible to compare immune cell phenotypes or total tumor cell numbers to detect a consolidation or processing error. Granted, there is always the possibility of finding differences between similar levels of the same biopsy specimen related to the natural geographic changes of the cells. Pathology comments added to the different samples are very important not only to explain those changes but also to have a retrospective record of what happened with a specific image analysis sample.

**FIGURE 3:**
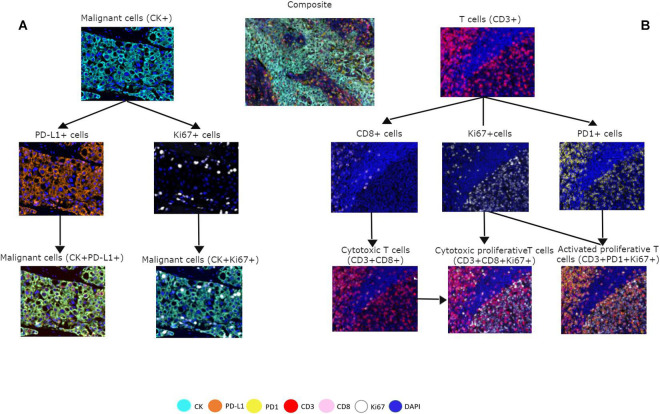
Multiplex immunofluorescence panel showing different cell phenotype co-localizations in tumor and immune cells from a non-small cell lung cancer sample. **(A)** Marker expression of malignant cells with cytokeratin (CK) and co-localization with PD-L1 + and Ki67 +. **(B)** Marker expression of CD3 + on immune T-cells, expression of CD8 + cells, expression of PD-1 + cells, and co-localization with CD3 + CD8 + for cytotoxic T-cells, Ki67 + CD3 + CD8 + for cytotoxic proliferative T-cells, and CD3 + Ki67 + PD-1 + for activated proliferative T-cells. The composite image with all the markers is localized in the upper center of the image.

**FIGURE 4:**
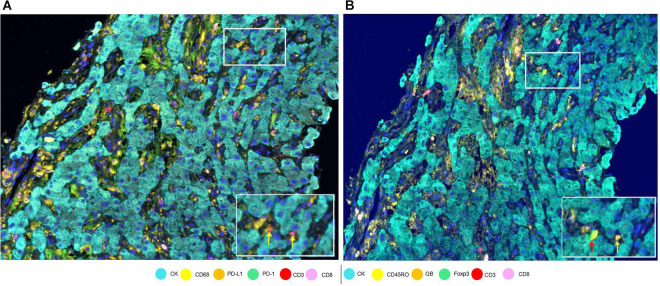
Marker expression in a hepatocellular carcinoma sample. **(A)** Low-magnification image with white-framed rectangles showing two CD3 + cells (indicated by yellow arrows) seen with high magnification at the bottom right of the panel. **(B)** Low-magnification image with white-framed rectangles showing the same two CD3 + cells with co-localizations: CD3 + Foxp3 + CD45Ro (indicated by a red arrow) and CD3 + CD45Ro (indicated by a yellow arrow) seen with high magnification at the bottom right of the panel.

**FIGURE 5:**
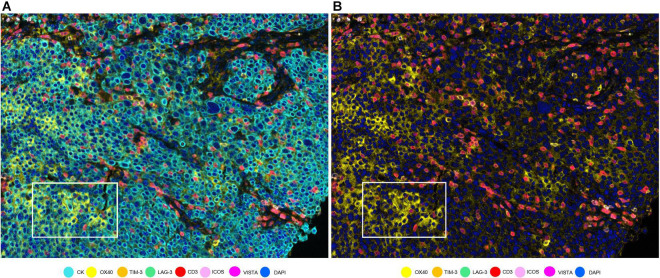
OX40 expression in tumor cells in a non-small cell lung cancer sample. **(A)** Composite with all the markers. White rectangle shows tumor cells with co-localization of cytokeratin (CK, cyan) and OX40 (yellow). **(B)** Composite with all markers except cytokeratin. The white-framed rectangle is the same area as in **(A)**.

To assess cell phenotypes according to the markers in a panel, we use the information given by the image analysis software about the marker expression of each individual cell according to their X and Y coordinates on the image. In this way, with the data consolidation, we can determine all the markers expressed by a single cell and, with this information, identify specific cell phenotypes. Commonly, many cell phenotypes can be identified according to markers in a mIF panel. Panels aimed to study lymphocytes can identify specific cell phenotypes (Figure 3), such as cytotoxic T-cells (CD3 + CD8 +), regulatory T-cells (CD3 + CD4 + FOXP3 +), memory T-cells (CD3 + CD45RO +), or T-cells expressing immune checkpoint markers, such as CD3 + PD-1 + or CD3 + PD-L1 + (Figure 4). The marker combinations are unlimited and, depending on the panel and markers, are able to show activation of markers, such as OX40 in tumor cells (CK + OX40 +) and rare cells, such as cytotoxic T-cells that express immune checkpoints (e.g., CD3, CD8, PD-1, and PD-L1; Figure 5).

**FIGURE 6:**
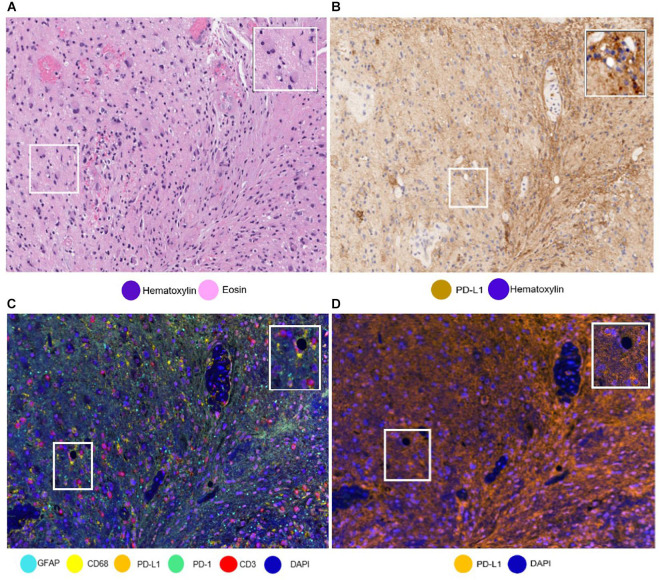
Same areas of a glioblastoma sample with different stainings. The glioblastoma sample stained with **(A)** hematoxylin and eosin and with **(B)** PD-L1 immunohistochemistry. It is important to note the challenge to differentiate immune cells from tumoral cells. **(C)** The glioblastoma sample stained using multiplex immunofluorescence (mIF). Using mIF allows us to differentiate lymphocytes from tumor cells (as seen in 3 CD3 + lymphocytes staining red and glial fibrillar acidic protein staining cyan). **(D)** mIF with only PD-L1-positive cells. White-framed rectangles in all the images highlight the same area of the sample, which is augmented in the rectangles on the upper right of each panel.

The availability of unlimited combinations of markers opened new ways to study tumor tissues, making the study of multiple markers possible. Additionally, while being a challenge when using standard methods, such as single IHC, differentiating the cell types that express these markers (e.g., tumor cells from immune cells) has also been made possible (Parra, 2018; Parra et al., 2019b). We have found this ability to differentiate the cells very useful in the study of PD-L1 in glioblastoma samples, because PD-L1 can be expressed by tumor cells, microglia, macrophages, and lymphocytes, with a wide range of patterns of tumoral morphology, making it very challenging to discriminate a cell with only single IHC (Figure 6) (Chen et al., 2018).

**FIGURE 7:**
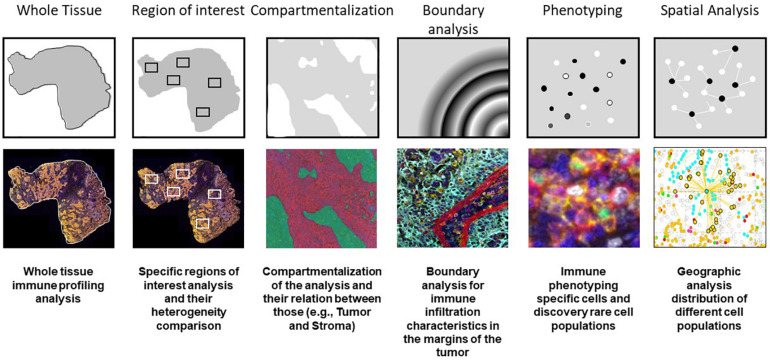
Examples of multiplex immunofluorescence capabilities for tissue immune cell phenotyping spatial analysis.

When we perform data consolidation, we can study not only the density of cell phenotypes but also the spatial placement of those cells in the tumor, allowing for the study of possible excitatory or inhibitory signals related by their proximity with the tumor cells or their neighbors. Phenotyping of cells *in situ* allows to establish those cells located close enough to interact with each other in immune activity. This approach is achieved using a different software, such as R-studio or SAS with the X and Y coordinates of each cell given by the image analysis software (Nagl et al., 2016; Lazarus et al., 2018). Other methods can be used, such as spatial metrics from the G-function (Barua et al., 2018) or infiltration analysis, to determine the number of objects or cells within a set range of an annotated region of interest (Kather et al., 2018) (Figure 7).

## Tumor Immune Profiling

In recent years, many studies have demonstrated the significance of tumor immune infiltrate densities, cell phenotypes, and spatial localization for the prediction of clinical outcomes, survival, and response to treatment (Pilla and Maccalli, 2018; Parra et al., 2020a). The direct simultaneous evaluation of immune tumor-related interactions and their spatial localization in a single tissue sample using multiplex techniques may allow a more accurate patient stratification for immunotherapy (Cascone et al., 2020; Provencio et al., 2020). A study that used multiplex IHC in head and neck squamous cell carcinomas showed a high infiltration of CD8 + T-cells and other T-helper type 1-associated immune infiltrates, indicating the presence of anti-tumor immunoreactivity. Furthermore, portion of these tumors exhibited the high myeloid cell infiltration profiles, and these tumors were associated with a poor prognosis. In the same study, the authors revealed that the response of pancreatic ductal adenocarcinomas to neoadjuvant vaccination therapy correlated with the grade of mono-myelocytic cell density and percentages of CD8 + T-cell exhaustion markers (Tsujikawa et al., 2017).

In the study of rare tumors, such as sarcomas, which in some cases may exhibit poor prognosis and adverse clinical outcomes (Dangoor et al., 2016), immune profiling has become a powerful tool in the characterization and understanding of tumor behavior. For example, immune profiling of Ewing sarcomas has demonstrated an association between higher densities of immunosuppressive M2 macrophages and a shorter event-free survival. Moreover, high frequency of T-cells and activated natural killer cells correlated with prolonged overall survival. Targeting macrophages, alone or in combination with other treatments, could be an interesting novel strategy for personalized medicine (Stahl et al., 2019). The rationale for immunotherapy in sarcomas is also explained by the presence of possible treatment targets, such as chromosomal alterations, or the cancer antigens resulting from genetic mutations. The presence of lymphoid tertiary structures and the rest of a naturally occurring immune infiltrate in sarcomas suggest that immunotherapy, such as cancer vaccines, adoptive cell therapy, and immune checkpoint blockade, may be feasible (Tseng et al., 2014). Because of this, we have found that analysis of the immune tumor microenvironment provides profound understanding of tumor behavior and novel treatment options.

Multiplex technologies provide unique sample-sparing analytical tools to characterize limited clinical tissue samples by allowing for *in situ* profiling and for the simultaneous profiling of multiple targets of interest (Hofman et al., 2019; Cascone et al., 2020; Provencio et al., 2020). We find the ability to study multiple markers in one specific cell especially useful in the study of cell densities, cell distribution, immune pathway marker expression in tumor cells, and recognition of new cell phenotypes, which can help explain the biological behavior of the immune system in relation to certain cancers. To accomplish this, we have created many mIF panels using six to eight markers to study cells according to their biological lineage (e.g., lymphoid or myeloid), immune activity (e.g., activated, pro-inflammatory, and regulatory), and presence of immune checkpoints (e.g., PD-1, PD-L1, B7-H3, B7-H4, and IDO-1). It is important to acknowledge that every mIF panel can and must be adapted to the purpose of the study and the type of sample being profiled.

One of the biggest advantages of mIF platforms is the ability to get a great deal of data from one slide, without the necessity of multiple sections as in IHC (Tan et al., 2020) or multiple staining followed by denaturalization steps as in sequential immunofluorescence staining (Wahlby et al., 2002). However, we do face certain challenges because tyramide signal amplification does not recognize the intensity of the antibody expression, which is conventionally used in the qualitative study of certain markers (Fedchenko and Reifenrath, 2014). Nevertheless, this same amplification has helped us to recognize weak signals that are difficult to evaluate in conventional IHC, such as arginase-1 in macrophages. Furthermore, the cell phenotyping (co-localization) tool is very handy to evaluate the immune score of PD-L1 in certain challenging malignancies, such as glioblastoma multiforme, in which differentiating tumor cells, lymphocytes, and macrophages is difficult. In cases like these, the combination of co-expression markers, such as glial fibrillar acidic protein, CD3, and CD68, can help to obtain very accurate results compared to single IHC.

## Conclusion

The advantages of mIF technologies are notable. The detection of multiple markers from a single tissue sample is both useful and necessary to provide comprehensive information about the cell nature, expression of prognostic markers, and even interactions between cells in the context of the tumor microenvironment. For imaging mIF with TSA, the ideal system is a scanner able to discriminate the different spectrums of the fluorophores used in a panel, giving us high-resolution images. As an example, the VectraPolaris scanner that combines the multispectral camera, the fluorescence cubes, and a resolution of 20 and 40x is able to capture high-quality images. Of course, other scanner systems can be used but probably those systems will be limited according to their assay-based specifications.

This technology is not exempt from limitations. For mIF, which currently uses tyramide signal amplification-based reagents, there is always the risk of tyramide overaction causing an umbrella effect. For this reason, it is important to evaluate the individual staining of each marker to recognize this possible effect during the optimization process. mIF methodology is also considerably more time consuming than a single bright field staining and subsequent imaging and digital pathology-related analysis. Also, there is the limitation of the number of antibodies in a panel which is basically the limitation in the spectrum of the fluorophores used. Eight markers per panel is the secure number of antibodies recommended in the workflow of the vendor in order to avoid any challenges in the optimization of new fluorophores and that can be discriminated easily by the scanner. However, new fluorophores can be tested and incorporated into the system. Nevertheless, a panel with less markers is more accurate and easily evaluated during the pathology image analysis compared with high plex technologies as imaging mass spectrometry or barcoding system that are used for exploratory purposes. In conclusion, the phenotyping process is complex, but mIF gives us tools to overcome many of the challenges that may arise. These tools and a deep understanding of immune cell protein expression and tumoral pathology are the key factors in the contribution of phenotyping and immune profiling to the study of tumor behavior and the development of new immunotherapies.

## Author Contributions

SH wrote most of the manuscript. FR, CL, RL, and IW contributed to the writing with their expertise on digital image analysis and immune profiling. EP developed the mIF technology in our laboratory and edited the manuscript according to his experience. All authors contributed to the article and approved the submitted version.

## Conflict of Interest

The authors declare that the research was conducted in the absence of any commercial or financial relationships that could be construed as a potential conflict of interest.

## Supplementary Material

Supplementary Figure 1 IPMN images with **(A)** tissue segmentation in different colors representing glass (blue), epithelium (red), and stroma (green) and **(B)** cell segmentation with cell nuclei (green center) and cell membrane (red border).
