# Supplementary figures and images for: Multiplex Immunofluorescence Tyramide Signal Amplification for Immune Cell Profiling of Paraffin-Embedded Tumor Tissues
Source: Front Mol Biosci. 2021 Apr 29;8:667067. doi: 10.3389/fmolb.2021.667067 (PMC8118604; doi:10.3389/fmolb.2021.667067)

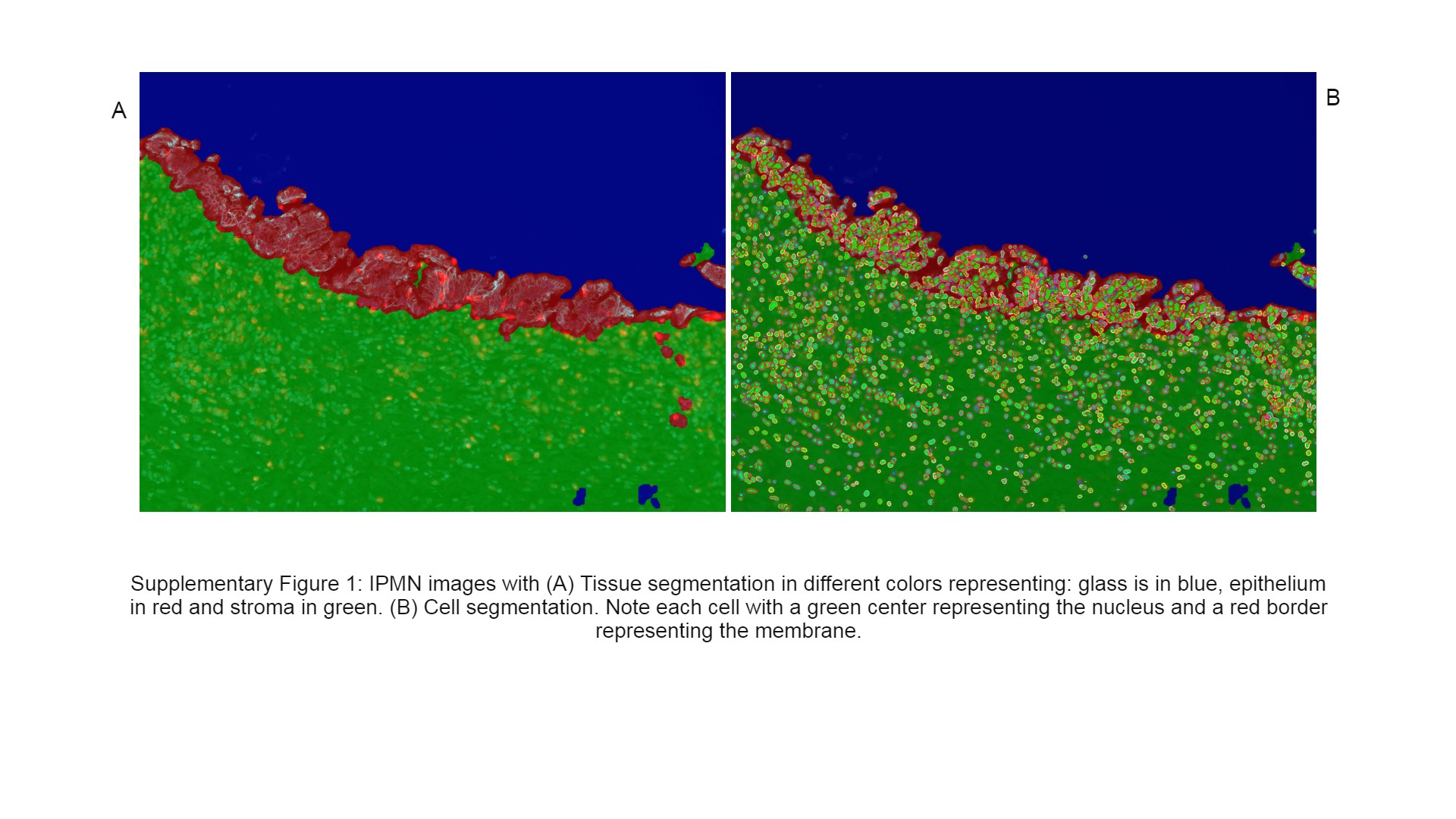

Supplement: Supplementary Figure 1 — IPMN images with (A) tissue segmentation in different colors representing glass (blue), epithelium (red), and stroma (green) and (B) cell segmentation with cell nuclei (green center) and cell membrane (red border). [file Image_1.JPEG]
